# A Recombinant Adenovirus Expressing P12A and 3C Protein of the Type O Foot-and-Mouth Disease Virus Stimulates Systemic and Mucosal Immune Responses in Mice

**DOI:** 10.1155/2016/7849203

**Published:** 2016-07-10

**Authors:** Yinli Xie, Peng Gao, Zhiyong Li

**Affiliations:** ^1^State Key Laboratory of Veterinary Etiological Biology, Key Laboratory of Grazing Animal Diseases of Ministry of Agriculture, Lanzhou Veterinary Research Institute, Chinese Academy of Agricultural Sciences, Lanzhou, Gansu 730046, China; ^2^College of Veterinary Medicine, Northwest A&F University, Yangling, Shaanxi 712100, China

## Abstract

Foot-and-mouth disease (FMD) is a highly contagious livestock disease of cloven-hoofed animals which causes severe economic losses. The replication-deficient, human adenovirus-vectored FMD vaccine has been proven effective against FMD. However, the role of T-cell-mediated antiviral responses and the mucosae-mediated antiviral responses induced by the adenovirus-vectored FMD vaccine was rarely examined. Here, the capsid protein precursor P1-2A and viral protease 3C of the type O FMDV were expressed in replicative-deficient human adenovirus type 5 vector. BALB/c mice immunized intramuscularly and intraperitoneally with recombinant adenovirus rAdv-P12A3C elicited higher FMDV-specific IgG antibodies, IFN-*γ*, and IL-4 cytokines than those in mice immunized with inactivated FMDV vaccine. Moreover, BALB/c mice immunized with recombinant adenovirus rAdv-P12A3C by oral and intraocular-nasal immunization induced high FMDV-specific IgA antibodies. These results show that the recombinant adenovirus rAdv-P12A3C could resist FMDV comprehensively. This study highlights the potential of rAdv-P12A3C to serve as a type O FMDV vaccine.

## 1. Introduction 

FMD is one of the most highly contagious viral diseases of cloven-hoofed animals that causes severe economic losses worldwide [1–3]. The causative agent, FMD virus (FMDV), belongs to genus* Aphthovirus*, family Picornaviridae, and consists of an approximately 8.5 kb single stranded plus-sense RNA genome that encodes a polyprotein. This polyprotein is subsequently cleaved by viral proteases leader (Lpro) and 3Cpro to produce structural and nonstructural proteins required for virus assembly and replication. The 3C protease cleavage products of the P1 region are structural proteins VP0, VP3, and VP1. One copy of each of the proteins VP0, VP1, and VP3 assembled into a protomer structure. Five protomers can assemble into a pentamer; then, 12 pentamers assemble into the final empty capsid structure. At last, the encapsidation of the plus-strand viral RNA accompanied by the maturation cleavage of VP0 to VP2 and VP4 forms the mature virion. At present, inactivated virus vaccines are widely used to prevent and control FMD [4, 5]. Although it is proved to be effective, there are many drawbacks of current inactivated vaccines, including the risks of inactivating the virus incompletely and of virus escaping from the manufacturing facilities. Moreover, it is difficult to distinguish those naturally infected animals from the vaccinated ones. Because of the limitations described above, some alternative vaccine strategies have been extensively studied. Among them, a recombinant, replication-defective human adenovirus type 5- (Ad5-) vector FMD vaccine was proved to be significantly efficient [6]. But most of these efforts were directed toward the induction of neutralizing antibody responses of vaccinated animals, while the T-cell-mediated antiviral responses and the mucosae-mediated antiviral responses were rarely examined [7, 8].

Although there is still some controversy about the role of cellular immunity in the protection of animals from FMD, specific T-cell-mediated antiviral responses have been demonstrated in cattle and pigs following vaccination [9–11] and it has been suggested that cellular immune response is important to clear the virus from persistently infected animals [12]. On the other hand, because aerosol transmission of FMDV is an important route of infection, induction of mucosal response is believed to be effective way against such infection. Moreover, the natural tropism of Ad5 for mucosal surfaces makes it an ideal choice as a mucosal vaccine delivery vector. In the present study, not only were the IgG antibody titer and cytokines detected in mice immunized intramuscularly and intraperitoneally, but also the IgA antibody titer was detected in mice immunized by oral and intraocular-nasal immunization.

## 2. Materials and Methods 

### 2.1. Plasmids, Cells, and Viruses

Adenoviral plasmid pAdEasy®-1 and shuttle vector pAdTrack-CMV were the gifts of Professor Shuhui Wang (Chinese Academy of Medical Science). Low passage human embryonic kidney 293 cells (HEK293 cells) were purchased from the Cell Bank of Chinese Academy of Sciences. Control adenoviruses (WtAdv) were persevered by our lab.

### 2.2. Animals

BALB/c mice are provided by the Lanzhou Veterinary Research Institute, Chinese Academy of Agricultural Sciences, and used following the national guidelines for the use of animals in scientific research.

### 2.3. Generation of Recombinant Adenoviruses

Using the plasmid pMD19-P12A3C preserved by our laboratory as a template, the polyprotein coding region of P1-2A and 3C viral protease were amplified and cloned into multiple cloning site (MCS) under control of cytomegalovirus (CMV) promoter in pAdTrack-CMV vector to generate pTrack-P12A-3C. The plasmid pAdEasy-1 containing most of the Ad5 genome was transformed into* E. coli *competent cells (Ad-BJ5183). Then, the recombinant vector pTrack-P12A-3C was linearized by digestion with* Pme *I and transformed into the same competent cells to produce pAd5-P12A-3C by homologous recombination with pAdEasy-1. The resulting vector plasmids pAd5-P12A-3C were linearized with* Pac *I and transfected into HEK-293 cells mediated by lipofectamine 2000 (Invitrogen) to generate the recombinant adenovirus. The recombinant adenovirus rAdv-P12A3C could be harvested when obvious cytopathic effect (CPE) and green fluorescence could be observed by fluorescence microscope. Recombinant adenovirus genome was extracted and purified by QIAamp® MinElute® Virus Spin Kit (QIAGEN) and the targeted gene P12A-3C was amplified with primers P12A3C-F and P12A3C-R, while the WtAdv was used for negative control. The recombinant adenoviruses were purified by ViraBind*™* Adenovirus Miniprep Kit (Cell Biolabs) according to the manufacturer's protocol and the adenovirus titer was monitored for virus titers by QuickTiter*™* Adenovirus Quantitation Kit (Cell Biolabs, Inc.).

### 2.4. Analysis of Expression of the Targeted Gene in HEK293 Cells

HEK293 cells were propagated in DMEM supplemented with 10% heat-inactivated FBS at 37°C. For protein expression experiments, cells were seeded into T-75 tissue culture flasks and infected with either rAdv-P12A3C or WtAdv at a multiplicity of infection (MOI) of 5 pfu/cell. All the media and the cells were harvested when most of the cells showed CPE; then, the media and cells collected were centrifuged at 1000 rpm for 5 minutes to pellet the cells. Cells pelleted were resuspended with DMEM and the virus was released by freeze/thaw cycles at −70°C and room temperature for three times. Finally, the virus supernatant was clarified by centrifugation at 1000 rpm for 10 minutes.

Western blot was used to detect the targeted protein; the virus supernatants clarified were separated by 12% SDS-PAGE and transferred onto a nitrocellulose membrane for 2 h at 200 mA. The membrane was blocked and then incubated with rabbit anti-FMDV polyclonal antibodies (1 : 1000 dilution) for 2 h. After several washes, the membranes were incubated with HRP-labeled goat anti-rabbit antibody (1 : 5000 dilution) and the bound antibodies were detected by chemiluminescence.

The virus supernatants clarified were also tested in indirect sandwich-ELISA (IS-ELISA). Briefly, the 96-well ELISA plate was coated with rabbit anti-FMDV polyclonal antibodies (1 : 1000 dilution) and incubated at room temperature overnight. The plate was washed and 50 *μ*L virus supernatants clarified were added and incubated at 37°C for 1 h. Then, the plate was successively incubated with the FMDV type O guinea pigs antiserum (1 : 1000 dilution), the anti-guinea pig IgG conjugated to horseradish peroxidase at 1 : 1000 dilution, and freshly prepared orthophenylene diamine/hydrogen peroxide substrate. The reaction was then stopped with 1.25 M H_2_SO_4_ and the absorbance was read at 490 nm using ELISA plate reader (BIO-RAD).

### 2.5. Intramuscular and Intraperitoneal Immunization in Mice

Thirty-six female BALB/c mice (aged 6 weeks) were randomly divided into three groups, with twelve mice in each group. All groups were intramuscularly (i.m) inoculated three times at an interval of 2 weeks. Group 1 was inoculated with a total of 5 × 10^9^ VP of WtAdv as a negative control. Group 2 was inoculated with a FMDV 100 *μ*L inactivated vaccine as a positive control, and group 3 was inoculated with a total of 5 × 10^9^ VP of rAdv-P12A3C. Another three groups were intraperitoneally (i.p) inoculated following what is mentioned above.

Blood samples were collected from each immunized mouse group at 0, 13, 27, and 41 days after the primary immunization for serological tests. At 42 days after the primary immunization, mice were sacrificed and splenocytes were harvested for enzyme-linked immunospot (ELISPOT) assay.

### 2.6. Detection of FMDV-Specific IgG Antibodies and Cytokines

The serum samples were tested in LPB-ELISA. Briefly, the 96-well ELISA plate was coated with rabbit anti-FMDV polyclonal antibodies (1 : 1000 dilution) and incubated at room temperature overnight. At the same time, 50 *μ*L of twofold serial dilutions of serum samples and 50 *μ*L predetermined FMDV antigens were added to the U-bottomed 96-well plate and incubated plate overnight at 4°C. The next day, the 96-well ELISA plate was washed 5 times with PBST and the antigen-antibody complex was added to the plate. The rest of the testing procedures were the same as the IS-ELISA. The absorbance was read at 490 nm using ELISA plate reader.

Using Mouse 1 × Lymphocyte Separation Medium (Dakewe Biotech Company) and RPMI 1640 medium, the splenocytes were isolated from the mouse spleens and blood then cultured in DMEM supplemented with 10% heat-inactivated FBS. The cultured IFN-*γ* ELISPOT assay was processed by using a mouse IFN-*γ* precoated ELISPOT kit (Dakewe Biotech Company). Briefly, the mouse splenocytes cultured were adjusted to a concentration of 2 × 10^6^ cells/mL and added 100 *μ*L per well to the 96-well ELISPOT plate which was precoated with anti-mouse IFN-*γ* antibody. The splenocytes were stimulated with the following materials: PMA+Ionomycin (positive stimulus, Dakewe Biotech Company), FMDV polypeptide: VVQAERFFKTHLFDWVTSDPF (provided by OUR LAB), and serum-free medium (SFM, negative stimulus). The final concentration of each stimulus was 10 *μ*g/mL. After incubation at 37°C and 5% CO_2_ for 20 h, crack the cells with cold ddH_2_O and the plate was successively incubated with biotinylated antibody, Streptavidin-HRP, and AEC coloring solution. At last, the whole plate was washed by tap water and spot-forming cells (SFC) were counted after being air-dried.

The cultured IL-4 ELISPOT assay was processed by using a mouse IL-4 precoated ELISPOT kit (Dakewe Biotech Company). The method was similar to the cultured IFN-*γ* ELISPOT assay.

### 2.7. Oral and Intraocular-Nasal Immunization in Mice

Thirty-two female BALB/c mice (aged 6 weeks) were randomly divided into three groups, with eight mice in each group. All groups were inoculated three times at an interval of 2 weeks. Group 1 was inoculated with 50 *μ*L rAdv-P12A3C by oral immunization. Group 2 was inoculated orally with 50 *μ*L WtAdv as a negative control, and group 3 was inoculated with 50 *μ*L rAdv-P12A3C by intraocular-nasal immunization. Group 4 was inoculated with 50 *μ*L WtAdv by intraocular-nasal immunization as another negative control. Blood samples were collected from each immunized mouse group at 0, 13, 27, and 41 days after the primary immunization for serological tests.

### 2.8. Detection of FMDV-Specific IgA Antibodies

The serum samples were tested by indirect ELISA. Briefly, the 96-well ELISA plate was coated with rabbit anti-type O FMDV polyclonal antibodies (1 : 1000 dilution) and incubated at room temperature overnight. The next day, the 96-well ELISA plate was washed and 50 *μ*L predetermined FMDV antigens were added to each well. The plate was incubated at 37°C for 1 h and washed before 50 *μ*L of twofold serial dilutions of serum samples was added to the U-bottomed 96-well plate starting with a 1 : 4 dilution. After incubation, 50 *μ*L anti-mouse goat IgA conjugated to horseradish peroxidase at 1 : 5000 dilution in PBST was added to the plate and incubated. Then, freshly prepared orthophenylene diamine/hydrogen peroxide substrate was added and incubated at 37°C for 15 min. The reaction was then stopped using 1.25 M H_2_SO_4_. The absorbance was read at 490 nm using ELISA plate reader (BIO-RAD).

### 2.9. T-Cell Proliferative Responses

Splenocytes were isolated from the oral and intraocular-nasal immunized mice as mentioned above, then cultured in DMEM supplemented with 10% heat-inactivated FBS, and adjusted to a concentration of 2 × 10^6^ cells/mL and added 100 *μ*L per well to the 96-well plate. The splenocytes were stimulated with the following materials: concanavalin A (positive stimulus), FMDV polypeptide, and PRMI 1640 (negative stimulus). The final concentration of each stimulus was 5 *μ*g/mL. After incubation at 37°C and 5% CO_2_ for 48 h, 40 *μ*L MTT was added to the U-bottomed 96-well plate and incubated plate at 37°C and 5% CO_2_ for 4 h. Then, 200 *μ*L DMSO was added to the U-bottomed 96-well plate. The absorbance was read at 490 nm using ELISA plate reader. The stimulation index (SI) was the ratio of stimulation group and nonstimulation group.

### 2.10. Statistical Analysis

Comparisons between two groups were accomplished by using an unpaired* t*-test. A significant difference was defined as *P* ≤ 0.05.

## 3. Results

### 3.1. Construction and Characterization of Recombinant Adenoviruses

**Figure 1:**
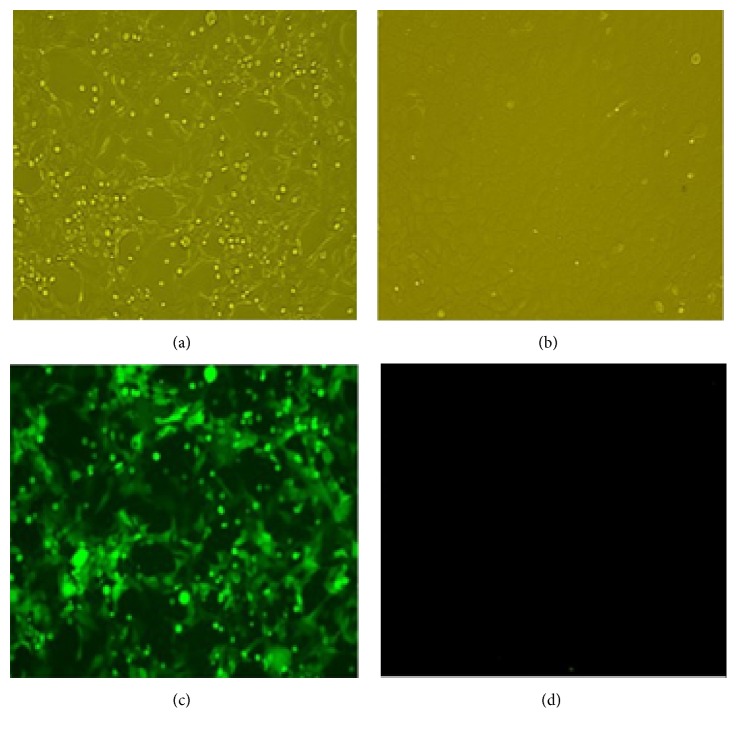
Observation of the CPE and fluorescence. (a) The HEK293 cells infected by the recombinant adenovirus; (b) normal HEK293 cells; (c) the green fluorescence of the infected HEK293 cells; (d) normal HEK293 cells under fluorescence microscope.

**Figure 2:**
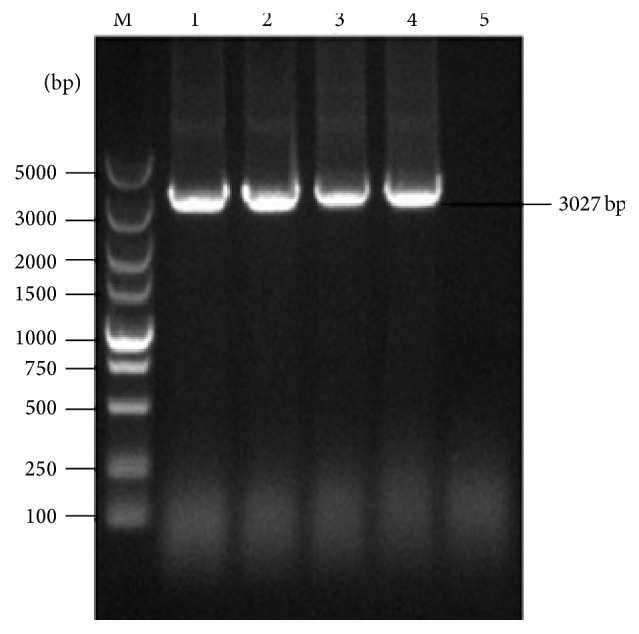
The stability identification of the inserted genes of the recombinant adenovirus by PCR. Lane M: the 5000 DNA marker; lanes 1–4: the amplified product of P12A-3C fragment of different passages (P3, P6, P9, and P12); lane 5: negative control.

Recombinant adenoviruses were obtained by the transfection of HEK293 cells with linearized pAd5-P12A-3C. The CPE (Figure 1(a)) and green fluorescent (Figure 1(c)) could be observed by fluorescence microscopy. The targeted gene P12A-3C was amplified with primers P12A3C-F and P12A3C-R from the recombinant adenovirus genome of different passages (P3, P6, P9, and P12) and a PCR product of 3027 bp in length was obtained which is consistent with the target gene P12A-3C, while nothing was obtained from the wild type adenovirus genome (Figure 2).

### 3.2. Detection of the Expressed Product of the Target Genes

**Figure 3:**
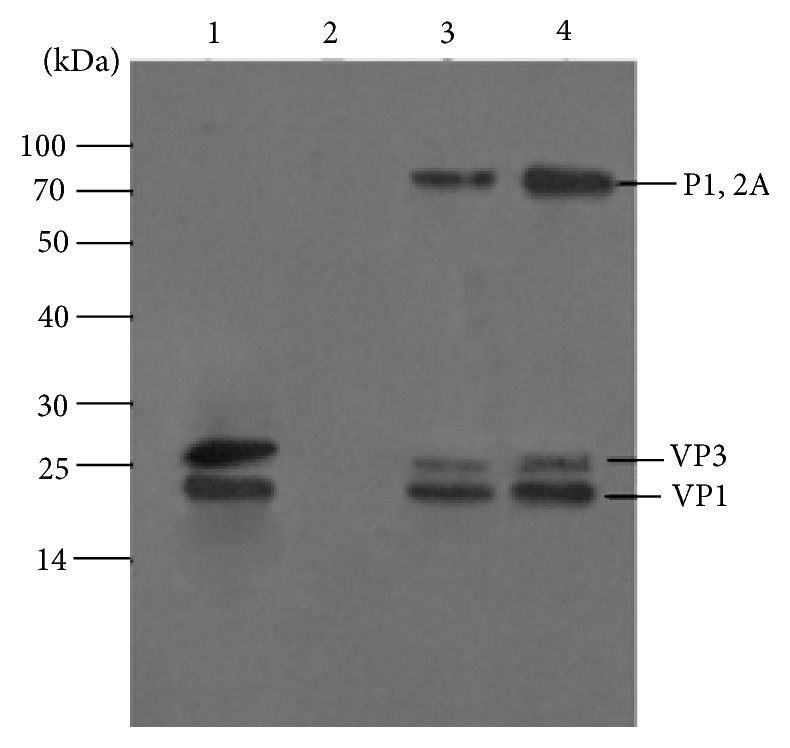
Western blot analysis of purified recombinant adenoviruses supernatant. Lane 1: BHK-21 cell lysates infected with FMDV; lane 2: WtAdv supernatant clarified; lane 3: the third-passage recombinant adenovirus supernatant clarified; lane 4: the sixth-passage recombinant adenovirus supernatant clarified. Protein molecular sizes (kDa) are indicated on the left.

**Figure 4:**
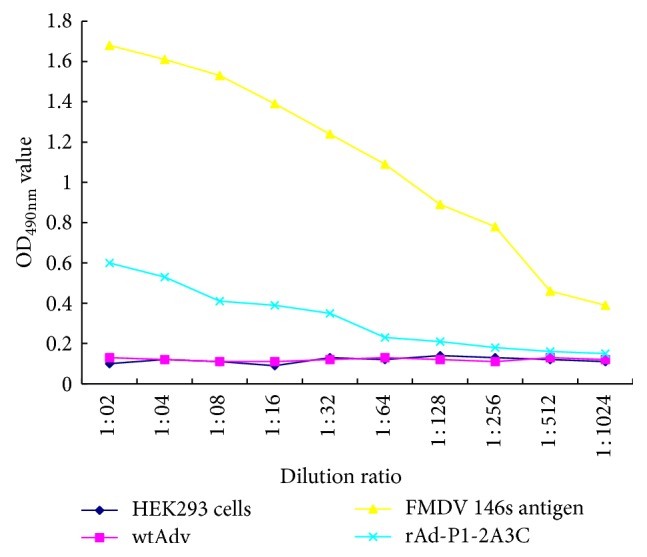
Detection of protein expression in rAd-P1-2A3C infected HEK293 cells by IS-ELISA. Supernatants clarified from HEK293 cells infected with rAd-P1-2A3C were processed at 36 h after infection. The data are presented as the mean of OD_490 nm_ for each dilution.

The expressed products were detected by Western blot (Figure 3) and IS-ELISA (Figure 4). The analysis of expression of FMDV structural proteins in Western blot showed bands of 23 kDa corresponding to VP1, bands of 27 kDa corresponding to VP3, and bands of 77 kDa corresponding to P1-2A in samples of rAdv-P12A3C and FMDV 146s antigen while nothing was detected in WtAdv sample. These results demonstrated that the recombinant adenovirus could efficiently express target protein in HEK293 cells.

### 3.3. Intramuscular and Intraperitoneal Immunization Induced High Anti-FMDV IgG Antibodies and High Cytokine Responses

**Figure 5:**
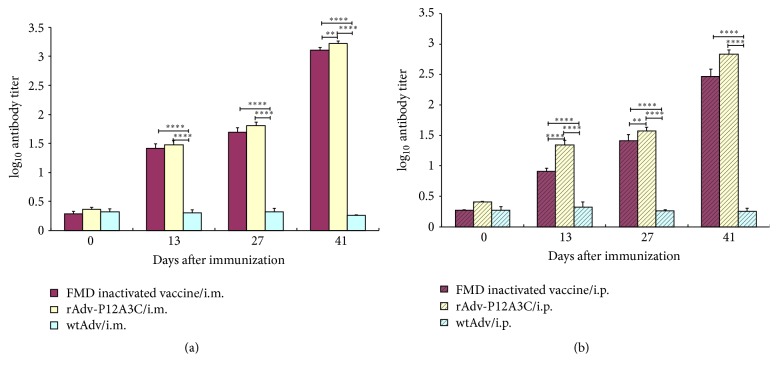
Detection of antibody titer in the immunized mice. (a) Vaccines given via i.m. route; (b) vaccines given via i.p. route. WtAdv is negative control; FMD inactivated vaccine is positive control. *P* values less than 0.05 were considered significant and, when represented by asterisks, are shown as follows: ^*∗*^0.05 ≥ *P* > 0.01; ^*∗∗*^0.01 ≥ *P* > 0.001; ^*∗∗∗*^0.001 ≥ *P* > 0.0001; ^*∗∗∗∗*^ *P* ≤ 0.0001.

**Figure 6:**
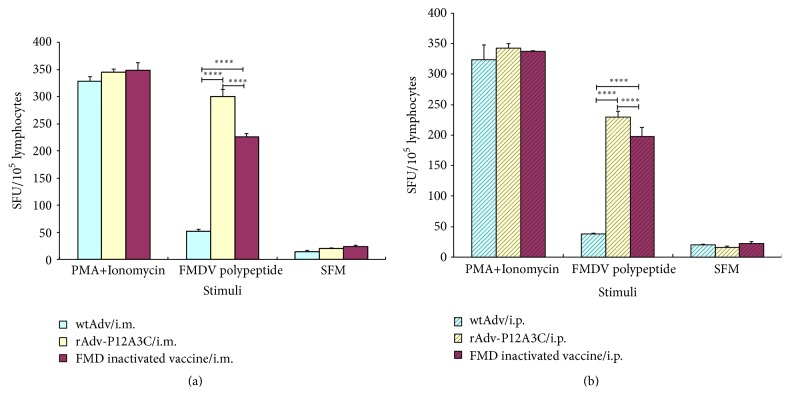
Detection of IL-4 in spleens of immunized mice by ELISPOT. (a) Vaccines given via i.m. route; (b) vaccines given via i.p. route. *P* values less than 0.05 were considered significant and, when represented by asterisks, are shown as follows: ^*∗*^0.05 ≥ *P* > 0.01; ^*∗∗*^0.01 ≥ *P* > 0.001; ^*∗∗∗*^0.001 ≥ *P* > 0.0001; ^*∗∗∗∗*^ *P* ≤ 0.0001.

The LPB-ELISA was proved to correlate well with virus neutralizing test (VNT) for measuring the serum neutralizing antibody titer of FMD vaccinated animals [13, 14], so we evaluated the specific anti-FMDV antibody response by LPB-ELISA. In the group rAdv-P12A3C and group FMD inactivated vaccine, all the mice were stimulated to produce antibodies against FMDV after the first inoculation, and the antibody titer was higher and higher after every immune (Figures 5(a) and 6(b)). After two immunizations, the antibody titer could reach a very high level and the antibody levels stimulated by rAdv-P12A3C were higher than those stimulated by FMD inactivated vaccine (Figures 5(a) and 6(b)), while the negative control group WtAdv was not stimulated to produce the specific antibody against FMDV in the whole process. This result suggests that the rAdv-P12A3C has the potential to serve as a type O FMDV vaccine.

**Figure 7:**
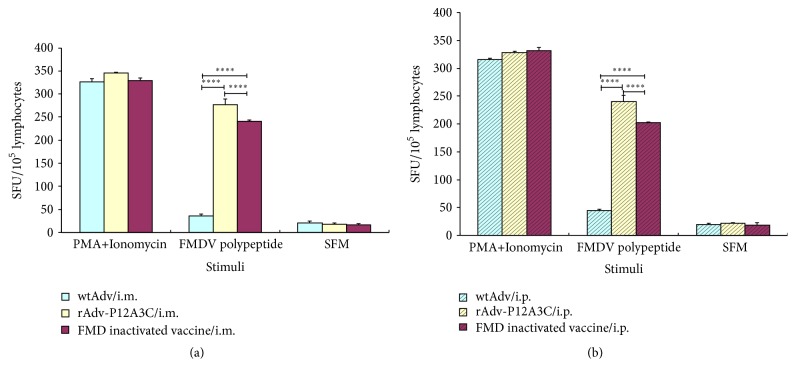
Detection of IFN-*γ* in spleens of immunized mice by ELISPOT. (a) Vaccines given via i.m. route; (b) vaccines given via i.p. route. *P* values less than 0.05 were considered significant and, when represented by asterisks, are shown as follows: ^*∗*^0.05 ≥ *P* > 0.01; ^*∗∗*^0.01 ≥ *P* > 0.001; ^*∗∗∗*^0.001 ≥ *P* > 0.0001; ^*∗∗∗∗*^ *P* ≤ 0.0001.

Splenocytes obtained from spleens of immunized mice via i.m. and i.p. routes were examined for IL-4 and IFN-*γ* to determine if cytokine responses are induced. The results are shown in Figures 6 and 7. In the groups stimulated with FMDV polypeptide, group rAdv-P12A3C induced higher expressions of IFN-*γ* and IL-4 than group FMD inactivated vaccine (*P* < 0.0001), while group WtAdv just induced very slow cytokine responses.

**Figure 8:**
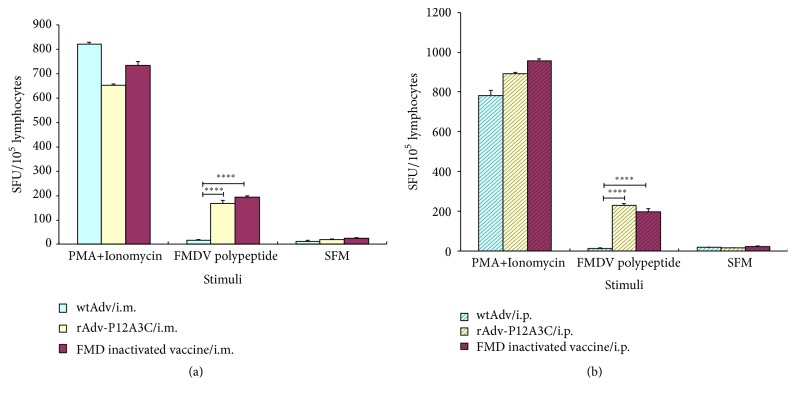
Detection of  IL-4 in blood of immunized mice by ELISPOT. (a) Vaccines given via i.m. route; (b) vaccines given via i.p. route. *P* values less than 0.05 were considered significant and, when represented by asterisks, are shown as follows: ^*∗*^0.05 ≥ *P* > 0.01; ^*∗∗*^0.01 ≥ *P* > 0.001; ^*∗∗∗*^0.001 ≥ *P* > 0.0001; ^*∗∗∗∗*^ *P* ≤ 0.0001.

**Figure 9:**
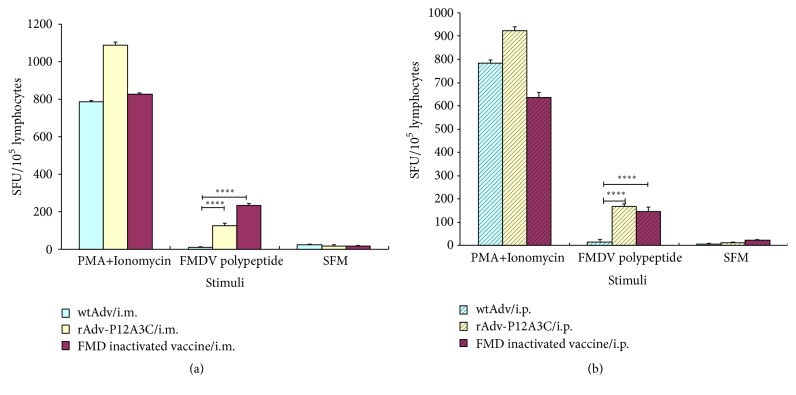
Detection of IFN-*γ* in blood of immunized mice by ELISPOT. (a) Vaccines given via i.m. route; (b) vaccines given via i.p. route. *P* values less than 0.05 were considered significant and, when represented by asterisks, are shown as follows: ^*∗*^0.05 ≥ *P* > 0.01; ^*∗∗*^0.01 ≥ *P* > 0.001; ^*∗∗∗*^0.001 ≥ *P* > 0.0001; ^*∗∗∗∗*^ *P* ≤ 0.0001.

Splenocytes obtained from the blood of immunized mice via i.m. and i.p. routes were examined for IL-4 and IFN-*γ* to determine if cytokine responses are induced. The results are shown in Figures 8 and 9. In the groups stimulated with FMDV polypeptide, group rAdv-P12A3C and group FMD inactivated vaccine induced high expressions of IFN-*γ* and IL-4, while group WtAdv just induced very slow cytokine responses.

### 3.4. Oral and Intraocular-Nasal Immunization Induced High Anti-FMDV IgA Antibodies

**Figure 10:**
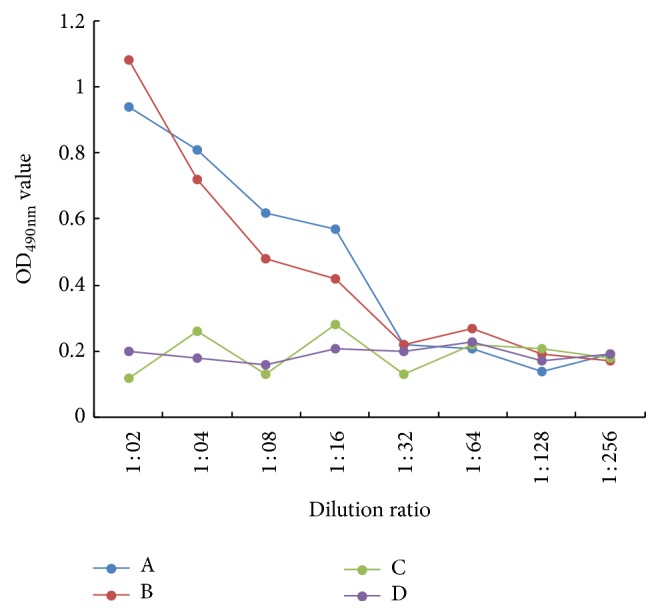
Anti-FMDV IgA antibodies detection by indirect ELISA. (A) rAdv-P12A3C given by oral immunization; (B) rAdv-P12A3C given by intraocular-nasal immunization; (C) WtAdv given by oral immunization; (D) WtAdv given by intraocular-nasal immunization.

The level of IgA antibodies against FMDV was determined by indirect ELISA method. The results are shown in Figure 10. In the groups inoculated with rAdv-P12A3C by oral and intraocular-nasal immunization, all the mice were stimulated to produce antibodies against FMDV after three times of inoculation, while the negative control group WtAdv was not stimulated to produce the specific antibody against FMDV in the whole process. This result suggests that the rAdv-P12A3C could induce mucosal IgA antibody against FMDV.

### 3.5. T-Cell Proliferative Responses

 The SI of rAdv-P12A3C given by oral immunization, rAdv-P12A3C given by intraocular-nasal immunization, WtAdv given by oral immunization, and WtAdv given by intraocular-nasal immunization, respectively, is 1.41, 1.36, 0.96, and 1.04. Groups immunized by rAdv-P12A3C can stimulate stronger T-cell proliferative responses than groups immunized by WtAdv.

## 4. Discussion

Vaccination of susceptible animals with inactivated vaccine had been adapted to control FMD. But because of the limitations of the inactivated FMDV vaccine, a novel vaccine that avoids the use of live virus is urgently required. The development of different types of recombinant FMDV vaccines has been extensively explored [15–17]. Although they are not as effective as the conventional inactivated FMDV vaccine, they may contribute to other valuable properties, mostly related to safety and type of immune response. Several studies have probed the protective effect of recombinant adenovirus expressing FMDV capsids protein of different serotypes; a study on a recombinant adenovirus carrying the P1 coding region of C type FMDV has been evaluated in cattle, showing that cattle vaccinated with this virus was partially protected [13]. Mayr et al. (1999) constructed a replication-defective Ad5 containing the coding region of FMDV empty capsid P1 polypeptide and viral protease 3C; pigs vaccinated demonstrated an antibody titer that reached a protective level of immunity. Furthermore, most of the research on new type vaccine of FMDV was stayed on the specific immunity antibody detection [7, 18]. So the purpose of this study is not only to build FMDV type O live carrier vaccine, expressing P12A and 3C protein of FMDV, but also to evaluate the effectiveness of the live carrier vaccine in both humoral and cellular immune responses.

Although there is a popular belief that protective immunity to FMDV is prevailing due to neutralizing antibodies, a T-cell response is also indispensable for effective immunity [19]. IL-4 plays a critical role in regulating the behavior of hematopoietic cells. In T-cells, it acts as a costimulant of cell growth and controls Th2 polarization. IFN-*γ* is a key cytokine produced primarily by T and NK cells which can facilitate host defense against intracellular pathogens [20]. In this study, both antibodies and cytokines were detected to evaluate the immune effect of the recombinant adenovirus. After three times of vaccination, rAdv-P12A3C could stimulate mice to produce high levels of antibodies, aiming at FMDV, rapidly and efficiently. And the levels of IL-4 and IFN-*γ* stimulated by rAdv-P12A3C were significantly higher than those stimulated by FMD inactivated vaccine. This result suggested that the rAdv-P12A3C could stimulate a strong T-cell response against FMDV. Stimulation of both cellular and humoral immune responses against FMDV might strengthen the protection of rAdv-P12A3C against FMDV.

As a member of Picornaviridae, FMDV could achieve its infection through the oral and respiratory mucosae. Therefore, delivery of vaccine to induce mucosal immunity is very important [21]. So we immunized the BALB/c mice with purified recombinant adenovirus rAdv-P12A3C by oral and intraocular-nasal immunization in order to determine whether the recombinant adenovirus could induce high FMDV-specific IgA antibodies or not. As a result, all the mice were stimulated to produce antibodies against FMDV after three times of inoculation. While the negative control group WtAdv was not stimulated to produce the specific antibody against FMDV in the whole process. This result suggests that the rAdv-P12A3C has the potential to against FMDV comprehensively.

Even though the first immunization could stimulate all the mice to produce antibodies against FMDV, we inoculated the mice another two times to ensure whether the immune effect of the recombinant adenovirus live carrier will affect the immune effect of the rAdv-P12A3C. We analyzed the change trend of antibody levels after every vaccination. And the antibody titer was higher and higher after every immunization (Figures 5(a) and 5(b)), demonstrating that the immunogenicity of adenovirus vector does not affect the immune effect of the rAdv-P12A3C, which is in agreement with Guo et al. [22]. Our experiment once again proved the practical application of adenovirus live carrier vaccine, as a new type of vaccine has a good development prospect.

## 5. Conclusion

Taken together, we expressed the capsid protein precursor P 1-2A and viral protease 3C of FMDV in replicative-deficient human adenovirus type 5 vector to produce capsid protein of the type O FMDV in HEK293 cells and demonstrated the immunogenicity of recombinant capsid protein in BALB/c mice. Our study highlights the potential of rAdv-P12A3C to serve as a type O FMDV vaccine.
